# Persistent hesitancy for SARS-CoV-2 vaccines among healthcare workers in the United Kingdom: analysis of longitudinal data from the UK-REACH cohort study

**DOI:** 10.1016/j.lanepe.2021.100299

**Published:** 2022-01-04

**Authors:** Christopher A. Martin, Katherine Woolf, Luke Bryant, Sue Carr, Laura J. Gray, Amit Gupta, Anna L. Guyatt, Catherine John, Carl Melbourne, I. Chris McManus, Joshua Nazareth, Laura B. Nellums, Martin D. Tobin, Daniel Pan, Kamlesh Khunti, Manish Pareek, UK-REACH Study Collaborative Group

**Affiliations:** a Department of Respiratory Sciences, University of Leicester, Leicester, UK; b Department of Infection and HIV Medicine, University Hospitals of Leicester NHS Trust, Leicester, UK; c University College London Medical School, London, UK; d Department of Nephrology, University Hospitals of Leicester NHS Trust, Leicester, UK; e General Medical Council, London, UK; f Biostatistics Research Group, Department of Health Sciences, University of Leicester, Leicester, UK; g Oxford University Hospitals NHS Foundation Trust, Oxford, UK; h Department of Health Sciences, University of Leicester, Leicester, UK; i Genetic Epidemiology Research Group, Department of Health Sciences, University of Leicester, Leicester, UK; j Population and Lifespan Sciences, School of Medicine, University of Nottingham, Nottingham, UK; k Diabetes Research Centre, University of Leicester, Leicester, UK

Healthcare workers (HCWs) in the United Kingdom (UK) have been prioritised in the SARS-CoV-2 vaccination agenda, including the ongoing booster programme.^1^ We previously reported that 23% of 11,584 HCWs who completed the baseline UK-REACH (UK Research study into Ethnicity And Covid-19 outcomes in Healthcare workers) cohort study questionnaire^2^ were hesitant about receiving a SARS-CoV-2 vaccine between 4^th^ December 2020 and 28^th^ February 2021. Vaccine hesitancy was more likely amongst certain ethnic minority groups and was associated with lower trust in employing healthcare organisations and in vaccines themselves. HCWs who were hesitant also reported concerns about vaccine safety and side effects, especially given the speed of vaccine development and roll-out, and expressed a desire to delay vaccination until more people had been vaccinated. As the vaccine programme progresses these concerns may lessen,^3^ however, the latest NHS England data show that around 15% of HCWs in some areas remain unvaccinated.^4^ To increase vaccine confidence and uptake for first, second and booster doses, we need to understand which HCWs are more likely to remain hesitant and why. This is particularly critical given the recent announcement that SARS-CoV-2 vaccination will be a mandatory requirement for UK HCWs from spring 2022.^5^ This leaves limited time to encourage HCWs to accept vaccination voluntarily, thereby lessening the potential deleterious effects of mandatory vaccination on staff morale and workforce retention.

Here, we report the persistence of hesitancy for first and second vaccine doses among UK HCWs, and the factors that predict persistent hesitancy. We analysed longitudinal data from the baseline and first follow-up UK-REACH questionnaire, the latter administered between 21^st^ April and 28^th^ June 2021 (53.1% of participants who completed the first questionnaire completed the follow-up). Our sample comprised UK HCWs who i) had completed both UK-REACH questionnaires, ii) reported being hesitant at baseline and iii) reported an incomplete SARS-CoV-2 vaccine schedule at baseline. Our outcome measure was remaining SARS-CoV-2 vaccine hesitant at follow-up. Participants were coded as remaining hesitant if they indicated hesitancy about their second dose at follow-up (if they had had a first dose at baseline) or for their first or second dose at follow-up (if they had had no doses at baseline). We constructed a logistic regression model to identify factors associated with remaining hesitant. We selected variables based on their association with vaccine hesitancy in our previous work and, to ensure results were relevant to policy, on which we felt employing healthcare trusts were likely to have data. The base model consisted of age, sex, ethnicity, job role and flu vaccination history (variables derived as previously^6^) on complete cases. To this model we then added variables representing i) trusted vaccine information sources; ii) information advocating against vaccination; iii) beliefs about the importance of vaccination and how well informed a participant felt about vaccines, to investigate their effect on persistent hesitancy.

**Figure 1:**
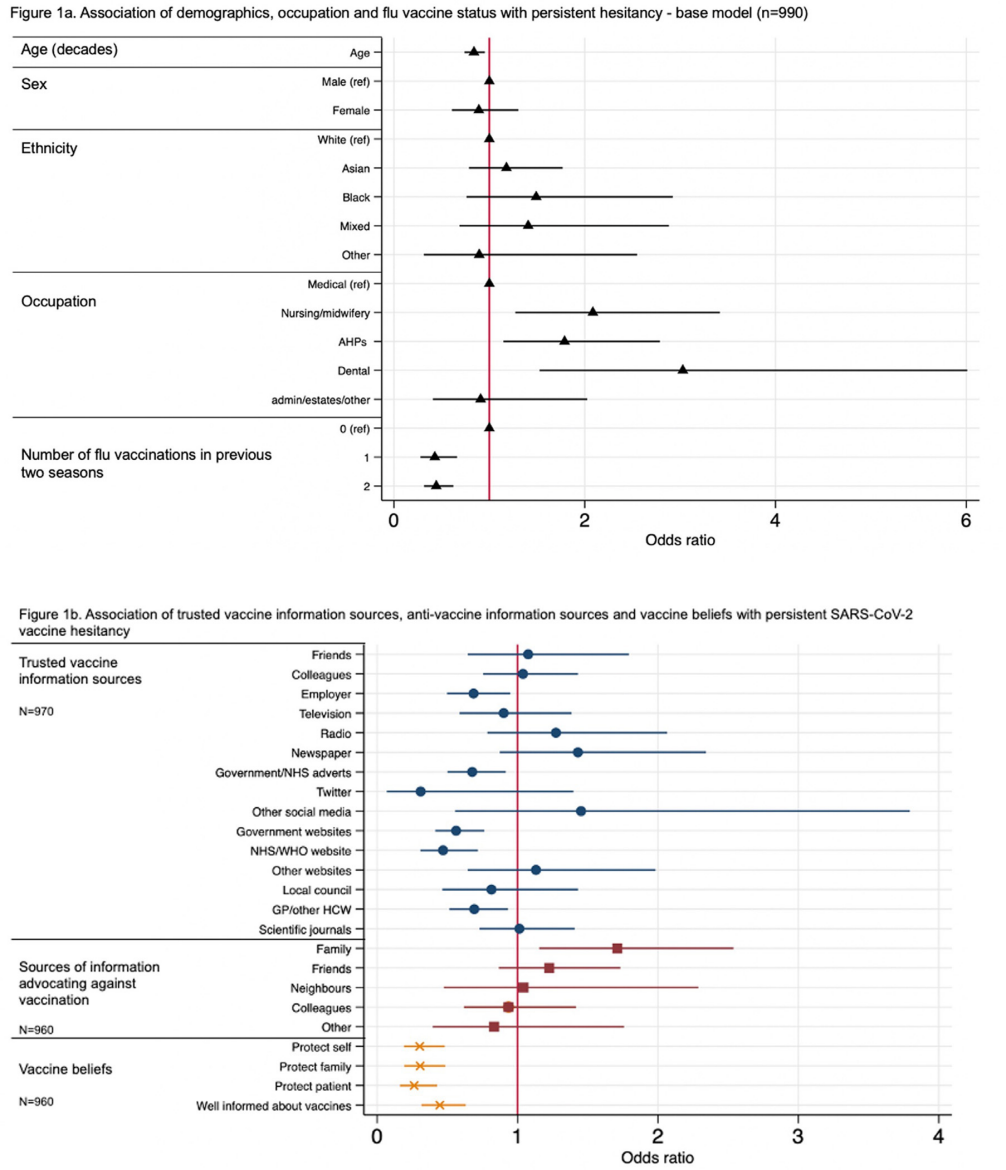
(a) shows the adjusted odds ratios for the association of covariates with an outcome of persistent SARS-CoV-2 vaccine hesitancy (adjusted for age, sex, ethnicity, occupation and flu vaccination status). Note that the allied health professionals category includes pharmacists, healthcare scientists, ambulance workers and those in optical roles and the nursing/midwifery category includes healthcare assistants and nursing associates. AHP – allied health professional, Ref – reference category. (b) shows the adjusted odds ratios for the association of i) trusted vaccine information sources, ii) sources of information advocating against vaccination and iii) vaccine beliefs with persistent SARS-CoV-2 vaccine hesitancy. These are adjusted for the variables in the base model. GP – general practitioner, NHS – National Health Service, WHO – World Health Organisation.

Results are shown in Fig 1a [n=990, n=275 (27.7%) remained hesitant] and Fig 1b (n=960 to 970). HCWs in nursing/midwifery roles (aOR 2.00, 95%CI 1.20 – 3.28), allied health professionals (including pharmacists, healthcare scientists, ambulance workers and those in optical roles; 1.79, 1.15 – 2.80) and dental roles (3.02, 1.53 – 6.01) were more likely to remain hesitant than those in medical roles. Those who had taken up influenza vaccination in the previous seasons were less likely to remain SARS-CoV-2 vaccine hesitant (0.43, 0.28 – 0.66 [for one influenza vaccination in previous two seasons], 0.45, 0.32 – 0.62 [for influenza vaccine uptake in both seasons]). Older HCWs were less likely to remain hesitant. There were no significant differences in risk of persistent hesitancy by sex or ethnic group.

HCWs who reported trusting vaccine information sourced from their employer (0.69, 0.50 – 0.95), Government/NHS adverts (0.68, 0.50 – 0.92), official websites (0.56, 0.41 – 0.76 [Government website], 0.47, 0.31 – 0.72[NHS/WHO website]), and their own GP/HCW (0.69, 0.51 – 0.93) were less likely to remain hesitant than those that did not trust these sources. Those that had been advised not to take the SARS-CoV-2 vaccine by their family were more likely to remain hesitant than those who had not (1.71, 1.15 – 2.54). HCWs who indicated that they agreed with statements regarding the importance of vaccines in protecting themselves (0.30, 0.19 – 0.48), their families (0.31, 0.19 – 0.49) and patients under their care (0.26, 0.16 – 0.43) were less likely to remain hesitant than those that indicated they did not agree with these statements. Those who indicated they felt well informed about SARS-CoV-2 vaccination were also less likely to remain hesitant (0.45, 0.32 – 0.63) than those who did not report feeling well informed.

A significant minority of HCWs in our sample were still experiencing vaccine hesitancy at follow up. In our previous work we highlighted trust (in employer, healthcare organisations and the Government) as a critical factor in predicting vaccine hesitancy.^6^ The results of the current analysis demonstrate that trust in these institutions is also important in determining whether hesitancy is likely to persist. Building trust amongst groups who are more likely to experience vaccine hesitancy may therefore represent a strategy of enhancing vaccine uptake. Importantly, we also demonstrate that having had family members advocate against vaccination increases the risk of persistent SARS-CoV-2 vaccine hesitancy. This highlights the importance of not only targeting interventions for improving vaccine uptake at HCWs, but also working to share messaging in their communities about the risks and benefits of vaccines to address concerns. Furthermore, our results indicate that any messaging designed to improve vaccine uptake and aimed at HCWs should emphasise the importance of vaccination for protection of HCWs, their families and their patients.

In summary, we have identified factors that might influence changes in vaccine hesitancy which should directly inform interventions aimed at improving vaccine uptake in HCWs and the wider community.
